# Phlebotomine sandfly ecology on the Indian subcontinent: does village vegetation play a role in sandfly distribution in Bihar, India?

**DOI:** 10.1111/mve.12224

**Published:** 2017-01-20

**Authors:** D. M. POCHÉ, R. M. POCHÉ, S. MUKHERJEE, G. A. FRANCKOWIAK, L. N. BRILEY, D. J. SOMERS, R. B. GARLAPATI

**Affiliations:** ^1^ Genesis Laboratories, Inc.WellingtonCOU.S.A.; ^2^ Genesis Laboratories, India Private LtdPatnaIndia

**Keywords:** Borassus flabellifer, Musa acuminata, Phlebotomus argentipes, banana plants, breeding sites, CDC light traps, dispersal, palmyra palm trees, vector control, visceral leishmaniasis

## Abstract

Visceral leishmaniasis (VL) is a disease that results in approximately 50 000 human deaths annually. It is transmitted through the bites of phlebotomine sandflies and around two‐thirds of cases occur on the Indian subcontinent. Indoor residual spraying (IRS), the efficacy of which depends upon sandfly adults resting indoors, is the only sandfly control method used in India. Recently, in Bihar, India, considerable sandfly numbers have been recorded outdoors in village vegetation, which suggests that IRS may control only a portion of the population. The purpose of this study was to revisit previously published results that suggested some sandflies to be arboreal and to rest on outlying plants by using Centers for Disease Control light traps to capture sandflies in vegetation, including banana plants and palmyra palm trees, in two previously sampled VL‐endemic Bihari villages. Over 3500 sandflies were trapped in vegetation over 12 weeks. The results showed the mean number of sandflies collected per trap night were significantly higher in banana trees than in other vegetation (P = 0.0141) and in female rather than male palmyra palm trees (P = 0.0002). The results raise questions regarding sandfly dispersal, oviposition and feeding behaviours, and suggest a need to refine current control practices in India and to take into account an evolving understanding of sandfly ecology.

## Introduction

Visceral leishmaniasis (VL) is a devastating disease that affects the poorest of the poor (Boelaert *et al.*, 2009). It is responsible for approximately 500 000 human infections and around 50 000 human fatalities each year, and is regarded as the second most deadly parasitic killer after malaria (Desjeux, 2001a, 2001b). The Indian subcontinent represents the largest VL focus in the world (Alvar *et al.*, 2012) and accounts for around 67% of reported VL cases (Chappuis *et al.*, 2007), in which *Leishmania donovani* (Kinetoplastida: Trypanosomatidae) is the incriminated pathogen (Singh *et al.*, 2006). In India, around 90% of VL cases occur in the impoverished state of Bihar (Singh *et al.*, 2006). Visceral leishmaniasis is transmitted through the bites of phlebotomine sandflies, small dipterans that rarely exceed 3 mm in length (Killick‐Kendrick, 1999), of which *Phlebotomus argentipes* (Diptera: Psychodidae) is the only incriminated VL vector on the Indian subcontinent (Dinesh *et al.*, 2000). Vector control through the use of insecticides may prove paramount in reducing sandfly numbers and subsequently reducing VL transmission in India. However, the organization and implementation of effective vector control strategies are hindered by a lack of reliable data regarding important aspects of sandfly ecology, including natural oviposition habitats and sugar sources (Warburg & Faiman, 2011).

*Phlebotomus argentipes* adults have been thought to be endophilic and endophagic, resting and feeding indoors, which is why indoor residual spraying (IRS) is the only vector control practice used in Bihar (Bern *et al.*, 2010). However, recent research suggests that at least a portion of *P. argentipes* may be exophilic and exophagic, resting and feeding outdoors. A study conducted over a 13‐month period in three Bihari villages by Poché *et al.* (2011) found that over 30% of the nearly 53 000 sandflies collected were found outdoors in vegetation. Of 288 blood‐fed sandflies found to be polymerase chain reaction‐positive to cytochrome *b* amplification, the majority (∼ 90%) were determined to have blood‐fed on either humans, cows (*Bos taurus*, *Bos indicus*) or domestic buffalo (*Bubalus bubalis*), and approximately 26% of blood‐fed sandflies were collected in outlying vegetation (Garlapati *et al.*, 2012).

The role of vegetation was also examined during a study in which over 5000 sandflies were collected from the canopies of palmyra palm trees (*Borassus flabellifer*) at up to 18.4 m above the ground (Poché *et al.*, 2012), a result which indicated that *P. argentipes*, although generally believed to be a weak flyer, is quite capable of vertical dispersal. The high number of sandflies distributed outdoors suggests that IRS may control only a portion of the sandfly population, given that its success depends on sandflies being endophilic (Coleman *et al.*, 2015).

The purpose of this study was to follow up on prior sandfly collections performed in outlying village vegetation (Poché *et al.*, 2011) and palmyra palm trees (Poché *et al.*, 2012) in order to provide further evidence of exophilic sandfly behaviour in Bihar. This involved the use of Centers for Disease Control (CDC) light traps to capture sandflies in outlying village vegetation and palmyra palm trees. The hypothesis was that sandflies would be found in peridomestic vegetation, as they were in the earlier studies (Poché *et al.*, 2011, 2012). This research may encourage managers to pursue means of control that explicitly target outdoor‐resting sandflies in order to complement the practice of targeting indoor‐resting sandflies through IRS.

## Materials and methods

### Study areas

The study was conducted in the Saran District, Bihar, India, approximately 30 km northwest of Patna. Two villages, Mahesia and Sutihaar, in which sandflies had been previously collected from outlying village vegetation (Poché *et al.*, 2011) and palmyra palm trees (Poché *et al.*, 2012), respectively, were selected.

#### Mahesia

The socioeconomic status of Mahesia (25°50′ N, 84°57′ E) is low (< US$500 per year per household). The majority of homes are constructed of thatch and brick and members of over 60% of households own livestock comprised mainly of cattle, buffalo, goats and chickens (Poché *et al.*, 2011). The village is highly agricultural and is surrounded by outlying vegetation including various vegetables and fruits. It is considered VL‐endemic, with the three most recent cases being reported in June (*n* = 1) and December (*n* = 2) 2014.

#### Sutihaar

The socioeconomic status of Sutihaar (25°51′N, 84°59′ E) is very low and is reflected in a high unemployment rate. The majority of homes are constructed of thatch and brick. Goats are the most abundant form of livestock. Wild rodents such as *Rattus rattus* and *Bandicota bengalensis* are also common. Outlying vegetation is highly abundant in this village, which is populated by over 500 palmyra palm trees, commonly used by villagers to produce palm wine (toddy). The village is VL‐endemic and numerous cases have been reported in recent years.

### Sandfly monitoring

Sandfly collection was performed between 23 September and 9 December 2015. After receiving permission from landowners, CDC light traps (Bioquip Products, Inc., Compton, CA, U.S.A.), powered by rechargeable 6‐V batteries, were positioned in outlying vegetation in Mahesia and in palmyra palm trees in Sutihaar. A protective cover was fitted to each trap to shield electronic components from rain and falling debris. The location of each CDC light trap was recorded using a handheld GPS (eTrex 30^®^; Garmin International, Inc., Olathe, KS, U.S.A.). Traps were set at around sunset and removed at around sunrise the following morning. Trap catches were then transported to Genesis Laboratories Pvt. Ltd in Patna and stored at −20 °C. Collected sandflies were separated from other insects, counted and morphologically identified according to sex and to one of the genera *Phlebotomus* or *Sergentomyia* (Diptera: Psychodidae). Only *Phlebotomus* sandflies were further identified to species level because this genus includes species incriminated as VL vectors.

#### Mahesia

In Mahesia, five CDC light traps were positioned in five outlying vegetation locations, with the light source positioned at approximately 1.0 m above the ground (Poché *et al.*, 2011). The vegetation type at each trap was noted and found to consist largely of maize and various fruits. The diversity of vegetation at each CDC light trap location is indicated in Table 1.

**Table 1:** Vegetation types present (X) at each of the five Centers for Disease Control light trap locations (trap nos. GLE‐1–GLE‐5) from which sandflies were collected during September–December 2015 in the village of Mahesia.

Vegetation type	Vegetation trap no.				
	GLE‐1	GLE‐2	GLE‐3	GLE‐4	GLE‐5
Mango (*Mangifera indica*)	X		X		
Guava (*Psidium guajava*)	X	X	X		X
Litchi (*Litchi chinensis*)	X	X	X		
Citrus (*Citrus* spp.)	X	X	X		
Maize (*Zea mays*)	X	X	X	X	X
Papaya (*Carica papaya*)	X				
Banana (*Musa acuminata*)	X				X
Teak (*Tectona grandis*)		X			
Jackfruit (*Artocarpus heterophyllus*)		X			
Sponge gourd (*Luffa aegyptiaca*)		X			X
Castor oil plant (*Ricinus communis*)			X		
Indian fig tree (*Ficus racemosa*)			X		
Palm tree (*Borassus flabellifer*)				X	
Beechwood (*Gmelina arborea*)				X	
Drumstick tree (*Moringa oleifera*)				X	
Bottle gourd (*Lagenaria siceraria*)					X
Bodhi tree (*Ficus religiosa*)				X	
Bamboo (Subfamily: Bambusoideae)				X	

#### Sutihaar

**Figure 1:**
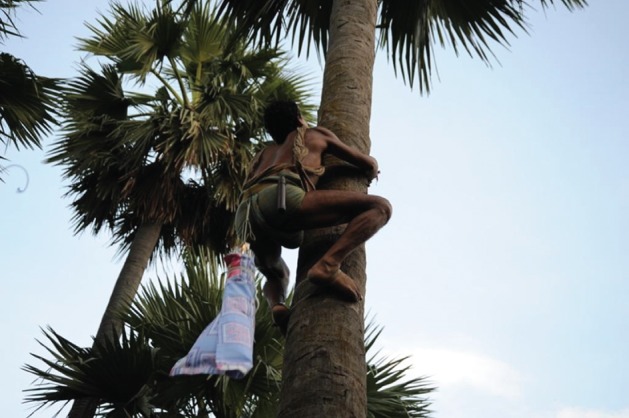
A hired climber setting a Centers for Disease Control light trap in a palmyra palm tree.

**Figure 2:**
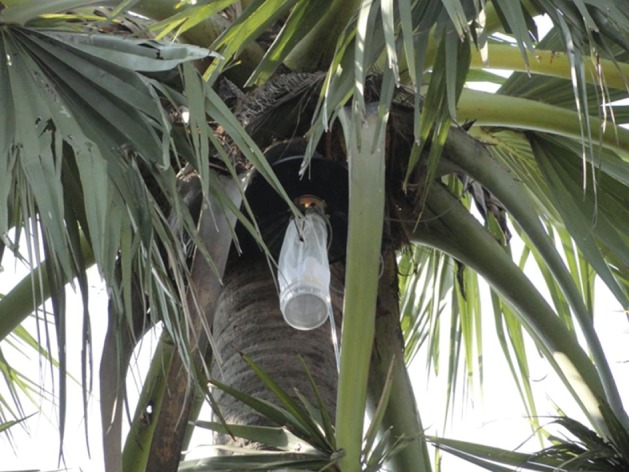
An operational Centers for Disease Control light trap placed in the canopy of a female palmyra palm tree (height: 11.3 m) on 23 September 2015 at 18.00 hours in the village of Sutihaar. The mechanical parts are shielded from rain and debris by a protective lid. The 6‐V battery that powers the trap is secured at the base of the branches.

In Sutihaar, 12 CDC light traps were positioned in the canopies of six male and six female palmyra palm trees (one CDC light trap per tree). The locations were selected based on the availability of the owners, who gave permission to set the traps. Once permission had been granted, two hired climbers set traps in the canopies of the trees at around 0.5–1.0 m away from the trunk (Figs 1 and 2). The heights of the palm trees ranged from 10.9 to 13.7 m (mean height: 12.2 m) (Table 2). Study personnel were present for the setting and collection of CDC light traps to ensure that traps were set in the canopies of the trees.

**Table 2:** Sex and heights of palmyra palm trees in which Centers for Disease Control light traps were set, and from which sandflies were collected, during September–December 2015 in the village of Sutihaar.

Palm trap no.	Sex of tree	Height, m
P‐1	Male	Unknown*
P‐2	Female	Unknown*
P‐3	Female	11.8
P‐4	Male	12.4
P‐5	Female	12.8
P‐6	Male	12.6
P‐7	Male	12.2
P‐8	Female	13.7
P‐9	Male	12.1
P‐10	Female	12.1
P‐11	Female	10.9
P‐12	Male	11.4

* Trees were burned in a village fire before measurements could be taken. Measurements were taken at the end of the study, after sandfly collection was completed.

### Data analysis

The mean number of sandflies per trap night in palms and vegetation was determined for each collection date. Differences in median values between the numbers of *P. argentipes* and *Sergentomyia* spp. per trap night collected from each village, between the numbers of sandflies per trap night collected from different vegetation types in Mahesia and, lastly, between the numbers of sandflies per trap night collected from male and female palmyra palm trees in Sutihaar, were assessed using a non‐parametric Wilcoxon rank sum test (*P* ≤ 0.05), with date of collection as a blocking variable.

## Results

### Sandfly captures

From 23 September 2015 to 9 December 2015 (12 weeks) a total of 3550 sandflies were collected over 204 trap nights. A total of 1490 *P. argentipes* (842 males, 648 females), 2058 *Sergentomyia* spp. (735 males, 1323 females), and two *Phlebotomus papatasi* (two males) were collected and morphologically identified.

#### Mahesia: outlying vegetation captures

**Figure 3:**
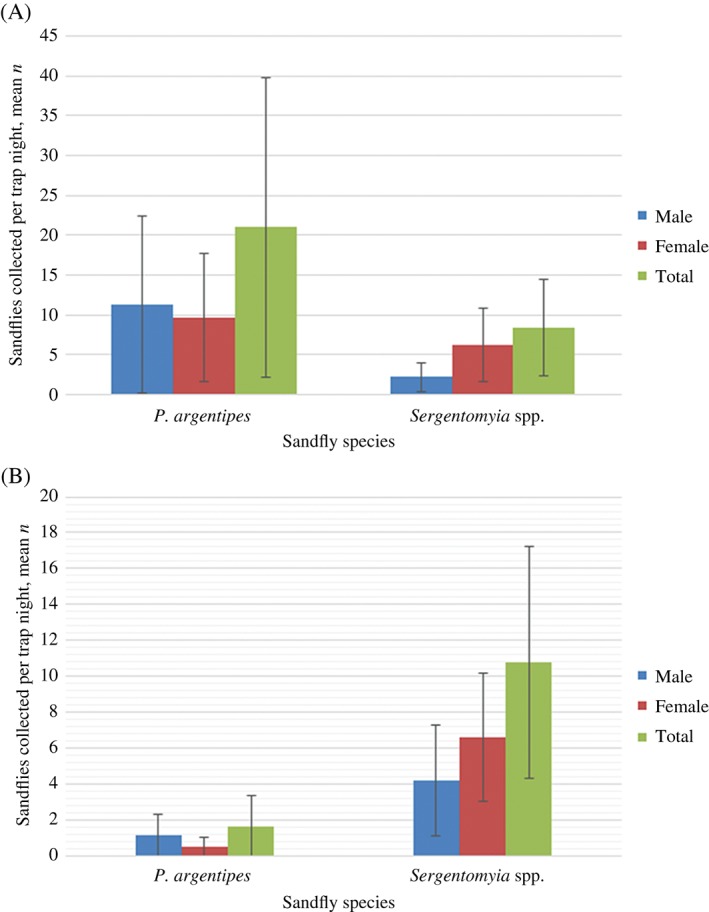
Mean numbers of sandflies collected per trap night (23 September to 9 December) in (A) five Centers for Disease Control (CDC) light traps positioned in outlying vegetation in the village of Mahesia (1259 Phlebotomus argentipes and 505 Sergentomyia spp.), and (B) 12 CDC light traps positioned in the canopies of 12 palmyra palm trees in the village of Sutihaar (231 P. argentipes and 1553 Sergentomyia spp.). Vertical bars represent the standard error. [Colour figure can be viewed at wileyonlinelibrary.com].

In total, 1764 sandflies were collected from outlying vegetation over 60 trap nights (29.4 sandflies/trap night) (Fig. 3A). Numbers of *P. argentipes* (20.98/trap night) collected in vegetation were markedly higher than those of *Sergentomyia* spp. (8.42/trap night), which supported data from the previous study (Poché *et al.*, 2011). Numbers of *P. argentipes* collected per trap night were significantly higher than those of *Sergentomia* spp. (*P* = 0.0029). The highest numbers were captured on 2 December (*n* = 363) and 25 November (*n* = 309). The lowest number was captured on 23 September (*n* = 17).

A high proportion of sandflies (∼ 63%) were collected from areas in which bananas (*Musa acuminata*) were present. A total of 1113 sandflies, amounting to an average of 46.4 sandflies per trap night, were collected from two traps positioned in areas with banana plants (trap nos. GLE‐1 and GLE‐5). This was noticeably higher than the cumulative number of sandflies (*n* = 651) and mean number of sandflies per trap night (18.1) collected from the three traps positioned in other areas in which bananas were not present (trap nos. GLE‐2, GLE‐3, GLE‐4) (Table 3). Additionally, the number of sandflies collected per trap night in locations with banana plants was significantly higher than numbers collected in other areas (*P* = 0.0141).

**Table 3:** Mean numbers of Plebotomus argentipes and Sergentomyia spp. per trap night collected during September–December 2015 in the village of Mahesia, in two Centers for Disease Control (CDC) light traps set in outlying vegetation containing banana plants and three CDC light traps set in other outlying vegetation.

Collection date	Sandflies collected in banana plants, mean *n*			Sandflies collected in other vegetation, mean *n*		
	*P. argentipes*	*Sergentomyia* spp.	Total	*P. argentipes*	*Sergentomyia* spp.	Total
23 September	1.5	3.0	4.5	0.7	2.0	2.7
30 September	0.0	3.5	3.5	18.0	2.3	20.3
7 October	5.5	31.5	37.0	9.0	1.7	10.7
14 October	17.0	12.5	29.5	14.7	6.0	20.7
21 October	9.5	14.5	24.0	2.7	4.3	7.0
28 October	26.5	38.5	65.0	8.7	13.3	22.0
4 November	20.0	17.5	37.5	17.7	8.3	26.0
11 November	28.0	18.5	46.5	16.0	8.7	24.7
18 November	10.5	1.0	11.5	11.3	2.3	13.7
25 November	126.0	6.5	132.5	9.3	5.3	14.7
2 December	164.5	14.0	164.5	11.3	4.0	11.3
9 December	0.5	0.0	0.5	40.7	2.7	43.3
Mean	34.1	13.4	46.4	13.3	5.1	18.1

#### Sutihaar: palmyra palm tree captures

A total of 1786 sandflies were collected from palm trees over 144 trap nights to give an average of 1.6 *P. argentipes*, 10.8 *Sergentomyia* spp. and 12.4 total sandflies per trap night (Fig. 3B). Additionally, numbers of *Sergentomyia* spp. collected per trap night were significantly higher than those of *P. argentipes* (*P* < 0.0001). The highest number of sandflies was captured on 11 November (*n* = 347) and the lowest number was captured on 9 December (*n* = 4).

In total, 1116 and 668 sandflies were collected from female and male palms, respectively (Table 4). Significantly more sandflies were collected per trap night in female trees than in male trees (*P* = 0.0002).

**Table 4:** Numbers of Plebotomus argentipes and Sergentomyia spp. captured in Centers for Disease Control light traps placed in male and female palm trees during September–December 2015 in the village of Sutihaar.

Collection date	*P. argentipes*, *n*		*Sergentomyia* spp., *n*		Total, *n*	
	Male trees	Female trees	Male trees	Female trees	Male trees	Female trees
23 September	0	2	29	42	29	44
30 September	3	5	53	61	56	66
7 October	1	32	33	138	34	170
14 October	8	2	52	109	60	111
21 October	4	6	73	74	77	80
28 October	3	5	66	97	69	102
4 November	13	26	69	162	82	188
11 November	34	43	111	159	145	202
18 November	6	4	13	35	19	39
25 November	3	12	83	69	86	81
2 December	3	14	5	18	8	32
9 December	1	0	2	1	3	1
Total	79	151	589	965	668	1116

## Discussion

The results of this study provide further evidence of consistent exophilic, nocturnal sandfly behaviour. As in previous experiments, in which over 7500 (Poché *et al.*, 2011) and over 5000 (Poché *et al.*, 2012) sandflies were collected from outlying vegetation in Mahesia and from palmyra palm trees in Sutihaar, respectively, sandflies were successfully collected from outlying vegetation and palmyra palm trees in the same two villages from September to December 2015.

Although the role of vegetation in determining sandfly distribution remains unclear, the present study suggests the relationship between sandflies and vegetation surrounding villages should be further evaluated. The current study reinforces the results of previous studies performed in the same area (Poché *et al.*, 2011, 2012) and also provides a more explicit evaluation of the locations of adult sandfly captures in an attempt to determine any preference for specific vegetation within Bihari villages. Although only female sandflies feed on blood (because they require nutrients for egg production), both male and female sandflies feed on natural sources of sugar such as vegetation sap (Killick‐Kendrick, 1999). Sugar‐rich sap is produced by banana plants (Pothavorn *et al.*, 2010) and palmyra palm trees (Barh & Mazumdar, 2008). In addition, according to (X. Chowdhury *et al.*, unpublished data, 2016) collected in Nepal, suggests that banana plants are preferred by *P. argentipes* collected in CDC light traps (M. L. Das, unpublished study, 2016). The high number of sandflies captured in banana plants may result from the nutritional quality of the sap produced. Additionally, organic material from banana plants and palm trees may provide substrate for *P. argentipes* oviposition. A comparison of collections by tree gender shows that a higher proportion of sandflies were collected from female palm trees (∼ 63%), a trend observed by Poché *et al.* (2012). The abundance of sandflies in female palmyra palm trees observed in the current study and previously by Poché *et al.* (2012) may be attributable to the excess pulp and mature fruit, and hence organic material, produced by female trees. Oviposition site surveys performed in Bihar and other parts of India focus largely on the collection of soil and organic material from in and around homes and cattle sheds (Ghosh & Bhattacharya, 1991; Kundu *et al.*, 1995; Singh *et al.*, 2008), historically yielding low numbers of sandflies. Outlying vegetation, including palm trees and banana plants, may provide sandflies with a variety of sources of organic material in which to oviposit. Although immature sandflies are rarely found during oviposition site surveys, in one study over 2000 sandfly larvae were recovered from forest floors in Panama (Hanson, 1961). Therefore, oviposition site surveys should not be limited to man‐made dwellings but should additionally include a variety of organic matter types surrounding villages. Narrowing the sizable gap in knowledge of sandfly oviposition sites, and hence of the locations of wild immature sandflies, should be a chief concern amongst managers and ecologists alike.

Additionally, the ability of sandflies to move vertically calls into question the belief that they are poor flyers. All of the palm trees from which sandflies were collected during this study were over 10 m in height. In a study currently in progress in Bihar, India, researchers observed *P. argentipes*, dusted with fluorescent powder, dispersing vertically up to around 6 m before disappearing from sight (D. M. Poché, Z. Torres, G. Garlapati, R. Poché; ‘Monitoring the short‐term movement of *Phlebotomus argentipes* in villages in Bihar, India’; unpublished study, 2016). This suggests that *P. argentipes* may be more capable of vertical and horizontal dispersal than previously suggested. Results of a previous mark–release–recapture study conducted by Killick‐Kendrick *et al.* (1984) indicated extended lateral mobility in *Phlebotomus ariasi* in France, with the maximum distances travelled from the release point being 600 m and 2.2 km in males and females, respectively. Recently, dispersal experiments in Israel determined the maximum travel distances of wild *Phlebotomus papatasi* to be 1.51 and 1.91 km in males and females, respectively (Orshan *et al.*, 2016). If *P. argentipes* possesses this level of dispersal capability, contrary to the prevailing belief that sandflies do not disperse far from breeding sites (Munstermann, 2004), further questions regarding disease transmission and vector competence in Bihar must be investigated. Assuming that greater dispersal capability increases the capacity of *P. argentipes* to transmit VL to previously non‐endemic areas, vector control at the village level may need to be re‐evaluated. Hence, if the movement of *P. argentipes* is not limited to the microhabitats available in individual villages and *P. argentipes* proves to be capable of moving laterally between neighbouring villages, vector control programmes will need to take this into account and to consider attempting sandfly control on a larger scale.

Therefore, it is recommended that future research should focus on the dispersal capability of P. argentipes.

It is interesting to note that *Sergentomyia* spp. outnumbered *P. argentipes* in palmyra palm trees in the current experiment. Numbers of *Sergentomyia* spp. (*n* = 1553) were markedly higher than those of *P. argentipes* (*n* = 231), a trend opposite to that observed by Poché *et al.* (2012). However, a study of vertical distribution performed in Kenya reported *Sergentomyia bedfordi* and *Sergentomyia antennata* as the only sandfly species to fly up to 11 m above the ground (Basimike *et al.*, 1989). It would be useful in future studies to attempt to determine if specific geographic and/or climatic variability is responsible for the differences between current and previous results.

## Conclusions

The results of the present study reinforce a need for integrated vector management incorporating novel control and preventative strategies to supplement the current practice of IRS. Perry *et al.* (2013) concluded that inhabitants in the majority of households within Bihari villages (∼ 95%) sleep outdoors for at least part of the year, particularly during the hot summer months. A study in which 93.7% of the 626 Bihari VL patients interviewed confirmed that they slept outdoors for part of the year drew similar conclusions (E. Hasker, R. Garlapati, R. Topno, A. Picado, M. Boelaert, R. Poché, P.K. Das; ‘Vector control for visceral leishmaniasis in India, are we targeting the right sandfly populations?’; unpublished study, 2016). Studies from India (Barnett *et al.*, 2005) and Nepal (Bern *et al.*, 2000) have concluded that sleeping outdoors is a significant risk factor for VL. Additionally, studies have found that bednet usage decreases in response to high evening temperatures (Kumar *et al.*, 2009; Claborn, 2010). Therefore, implementing an integrated vector control programme that combines a control strategy based on the resting sites of adult sandflies (IRS) with one dependent on the host and oviposition site preferences of sandflies (systemic insecticides) is encouraged. Previous studies from India involving *P. argentipes* (Ingenloff *et al.*, 2012; Poché *et al.* 2012) and Kenya involving *Anopheles* spp. (Diptera: Culicidae) mosquitoes (Poché *et al.*, 2015) have demonstrated the potential for the systemic insecticide fipronil to eliminate blood‐feeding vectors feeding on cattle and rodents and larvae feeding on excreted faeces. Additionally, if advantageous data regarding oviposition sites in Bihar become available, explicit larval control should be administered. Hence, the results of the present study raise questions regarding sandfly dispersal, oviposition and feeding behaviours and suggest a need to refine current control practices in India in a manner that takes into account an evolving understanding of *P. argentipes* ecology.
